# The Role of ChatGPT in Job Crafting: A Study of IT Professionals in Pakistan

**DOI:** 10.3390/bs16050655

**Published:** 2026-04-26

**Authors:** Seema Gul, Sajeela Rabbani, Aqsa Jaleel

**Affiliations:** 1 General Studies Department, College of Sciences and Humanities, Prince Sultan University, Riyadh 12435, Saudi Arabia; sgul@psu.edu.sa; 2 Iqra Business School, Iqra University Islamabad Campus, H-9, Islamabad 44000, Pakistan; 3 Riphah School of Leadership, Riphah International University, Islamabad 44000, Pakistan; aqsa.jalil@yahoo.com

**Keywords:** ChatGPT, job crafting, work-related curiosity, work engagement, information technology

## Abstract

The wake of artificial intelligence (AI) tools has witnessed a lot of changes at workplaces. Job crafting (JC) has also embraced the predictive quality of using AI tools such as ChatGPT. Drawing on Conservation of Resources theory, this study was conducted as an effort to understand the role that ChatGPT plays in job crafting by enhancing work engagement (WE) in the presence of work-related curiosity (WRC). Time-lagged data from 314 employees from the information technology (IT) sector was used to test the relationship by using partial least square structural equation modeling. The results showed that ChatGPT and job crafting are linked to each other in the presence of work engagement. The results further showed that WE mediated and work-related curiosity moderated the relationship between ChatGPT and job crafting. These results are instrumental in understanding the significance of AI adoption in business and can be used as a potential tool for crafting jobs toward other work-related outcomes. The research holds significance for mangers and policymakers of the IT sector in terms of establishing AI adoption to Predict positive behaviors in employees, and it also highlights future avenues.

## 1. Introduction

Technology has long been instrumental in shaping workplace practices and employee outcomes. In recent years, generative artificial intelligence (GenAI), particularly ChatGPT, has rapidly transformed how employees interact with digital systems (De Angelis et al., 2023). It can generate human-like responses across various media formats (codes or text) that emulate human searching behavior (Huh et al., 2023). Employees widely utilize it in their professional tasks and everyday work life, improving their productivity (Baidoo-Anu & Ansah, 2023), communication (Seetharaman, 2023), and interaction with work-related materials (Jauhiainen & Guerra, 2023). Bang and Park (2023) emphasized that ChatGPT-generated content in corporate environments can improve employee creative writing, language translation, learning experiences, and productivity due to its speed and ability to generate original data (Javaid et al., 2023).

Even though ChatGPT is becoming more popular in the workplace, empirical studies devoted to its effect on the work results of employees are scarce (Wang et al., 2024). One such domain unexplored fully is job crafting. The job crafting literature has not clearly described the use of AI-based conversational systems use as job resources to stimulate employees to redesign their work (Rice et al., 2024). Job crafting can be described as the way in which employees make personal adjustments to their work, such as modifying tasks, relationships, and cognitive aspects of the job (Tims & Bakker, 2010). Although the research has covered job crafting in the framework of traditional job resources, i.e., autonomy, feedback, or leadership support, a gap in knowledge exists regarding the ways in which new technologies, namely generative and interactive systems, such as ChatGPT, may serve as new cognitive resources supporting job crafting (Wang et al., 2024). Traditional technologies are more of a supplement to fixed processes or information retrieval, but ChatGPT is interactive, adaptive, and generative. It is able to formulate new ideas, formulate complex knowledge, and even imitate collaborative conversation in real time (Wang et al., 2024). These characteristics suggest that ChatGPT can influence efficiency, as well as motivation, perceived competence, and readiness to actively adjust roles of employees (Jauhiainen & Guerra, 2023).

The majority of AI studies have focused on technology adoption models, emphasizing why employees embrace AI systems, but offering limited insight into how AI use translates into proactive work behaviors such as job crafting. Given the nascent stage of this technology, identifying the mechanisms through which ChatGPT shapes employee motivation and behavior is therefore of paramount importance (Vrontis et al., 2023). In this regard, work engagement (WE) may serve as a critical mediator between the use of AI-related chatbots, such as ChatGPT, and employee job crafting. WE is characterized by vigor, dedication, and absorption in work tasks and is essential for employees when it comes to taking proactive steps in shaping their job roles (Attridge, 2009). Based on the Conservation of Resources (COR) theory, we frame ChatGPT as a novel digital resource that facilitates resource gain (e.g., reduced cognitive strain and enhanced perceived control), thereby fostering stronger work engagement. To fill this gap, the current research is based on the Conservation of Resources (COR) theory (Hobfall et al., 2018). The COR theory assumes that people seek to acquire, maintain, and accumulate prized resources, and resource acquisition leads to positive psychological conditions and prosocial behaviors. In this case, ChatGPT may be imagined as a new digital tool in the form of cognitive support, improved information access, and lessened mental load. Although it can bring about resource gains (e.g., efficiency and perceived control), it can also cause resource threats such as less autonomy, over-reliance, or deskilling. Job crafting is therefore not something that happens automatically; this is likely to happen when employees feel that ChatGPT empowers them and not that it restricts their agency. This contradiction raises the question of when and how the use of ChatGPT can result in engagement and proactive job redesign.

According to Sahab and Haqbeen (2024), AI tools are also key facilitators of WE. This not only improves job satisfaction and performance but also fosters a more innovative and adaptive workforce and ensures organizational success. By focusing on enhancing WE through AI support, personality factors help employees thrive, leading to improved job satisfaction, performance, and overall organizational success. In this regard, work-related curiosity also influences the relationship between employee’s use of ChatGPT and WE through certain boundary conditions of moderation (Agarwal & Gupta, 2018). Employees who exhibit a higher level of curiosity are more inclined to explore and utilize AI tools like ChatGPT effectively, enhancing their engagement with work tasks. The current study seeks to bridge these gaps and to contribute to both theory and practice in the information technology sector. Accordingly, we investigate the effects of employees’ ChatGPT usage on job crafting through enhanced work engagement in response to recent calls for research on AI-enabled work processes (Cardon et al., 2023; Marimon et al., 2024).

### 1.1. Theory and Hypotheses

Job crafting refers to the active process by which employees change tasks, relationships, and perceptions of their work to better suit the job according to personal strengths, preferences, and needs (Demerouti, 2014; Tims & Bakker, 2010). Past research indicates that job crafting is influenced by individual factors, social factors, and the organizational factors. Personal factors like resentment of injustice (Bipp & Demerouti, 2015) and developmental requirements require resiliency (Petrou et al., 2012), which also encourages employees to alter their work. Relational crafting is a type of negotiation between people, resources, and the wider social settings (Harju et al., 2023; MacGill, 2019), and Mudde (2022) focuses on craft knowledge in a relational, materialistic environment. The term cognitive crafting refers to reimagining the meaning of one’s work, and it can be both organization-centered and self-centered (Slemp & Vella-Brodrick, 2014). Combined, these studies indicate the intricate interaction of psychological and contextual factors on job crafting behaviors (Tims et al., 2013).

The use of ChatGPT is likely to produce work engagement, which in turn encourages employees to invest more resources in job crafting through the facilitation of resource gain, including perceived competence and control. Work engagement (WE), which is a dynamic state of commitment and absorption, is an inflated condition in which employees have the psychological capabilities needed to take proactive action in the redesign of their jobs. Notably, engagement, job crafting, and curiosity are conceptually different constructs. Work engagement refers to a state of mind described as being vigorous, dedicated, and absorbed (Attridge, 2009). In comparison, job crafting exhibits behavioral agency, the actual changes in work, tasks, relationships, or work meaning by employees (Tims & Bakker, 2010). In this respect, we hypothesize that the use of ChatGPT facilitates job crafting by increasing engagement.

### 1.2. ChatGPT and Job Crafting

Technology can help organizations to optimize processes and enhance productivity (Saleem & Malik, 2023). The idea of AI crafting has now been created in the setting of AI integration; it emphasizes how employees voluntarily adjust their jobs in response to the technologies. Although the adoption of AI can lead to positive results on the individual level, its introduction can be seen as a threat, and the employees can begin to design their jobs to get the meaning and control back (Meng et al., 2023). ChatGPT aids in the automation of duties, it enhances productivity, and it optimizes writing (Rathore, 2023), and it has accumulated millions of users within a very short time (Boschee, 2023). Having advanced language processing, it is able to produce text, and it can even simulate human-like conversations. Despite the issues with privacy and job loss, it is possible that ChatGPT can make workplaces more productive and enhance the quality of professional work in any industry. Indicatively, research has noted the effect of its use in terms of productivity improvement and writing quality. Although this has occurred, there is a need for more studies to determine what its wider organizational ramifications could be (Puri & Baskara, 2023). ChatGPT is used by IT workers for routine tasks such as knowledge augmentation, problem-solving, and content generation in their respective jobs. This type of use directly influences job crafting because it serves as a resource and brings autonomy to workers, along with flexibility. Relational crafting involves negotiation among people, resources, and complex settings; cognitive crafting pertains to reimagining the work’s purpose and meaning; while task crafting reflects changes and initiatives in one’s job tasks in terms of number and scope (Wrzesniewski & Dutton, 2001). We argue that task crafting is affected by the use of ChatGPT through changes in workflow and design (Wrzesniewski & Dutton, 2001). Since AI enhances critical capabilities, using ChatGPT might help employees reconstruct their jobs cognitively, feel competent, and re-frame the meaning of work, leading to cognitive crafting. It may also act as a cognitive resource that improves communication quality and frees up time for strategic partnership (Mollick & Mollick, 2023), thus shaping relational crafting. Hence, we argue that using ChatGPT brings three types of crafting jobs as depicted in the following hypotheses:

**H1a:**  *The use of ChatGPT is positively related to task crafting.*

**H1b:**  *The use of ChatGPT is positively related to cognitive crafting.*

**H1c:**  *The use of ChatGPT is positively related to relational crafting.*

### 1.3. ChatGPT and Work Engagement

Work engagement is an affirmative state in which workers are dedicated, fulfilled, and ready to absorb work-related challenges (Mazzetti et al., 2023, Shimazu et al., 2010). Unlike organizational commitment, it represents work-related vigor that enables workers to focus on the given task. Studies have shown that WE is dependent on job resources such as autonomy and supervisory support, as well as on personal resources like hope, optimism, and self-efficacy (Hakanen et al., 2024). The major predictors related with work are supervisory support, characteristics of job, and certain leadership behaviors (Tanuwijaya et al., 2022). Individual-level influencers include demographics, job nature, and psychological states, as well as training, development initiatives, and job simulations (Chandani et al., 2016). Recent studies have proposed that use of AI tools such as ChatGPT has marginally influenced the engagement level of individuals with their work (Barbas et al., 2023; Kumar & Upadhyay, 2023). This has also been tested in education, where it tends to enhance student engagement and their autonomy and motivation for their projects, as well as enhance their critical thinking (Abdelmagid et al., 2024; Zhao et al., 2023). For instance, it is a good predictor of performance across all other determinants of job performance (LePine & Van Dyne, 2001), such as agility performance (Liu & Chen, 2021), as well as innovation at workplace (Rogoza et al., 2024).

Studies have shown that curiosity predicts job performance incrementally, above and beyond traditional cognitive and non-cognitive predictors (Stoll, 2020). These findings suggest that curiosity is a good predictor and explanation of work-related behaviors (Mussel, 2022). The impact of AI adoption on job crafting within organizations has also been studied (Li et al., 2024). The available evidence shows that AI adoption will lead to a number of forms of job crafting by employees and managers in organizations (Lee & Park, 2023). AI affects job crafting particularly when people perceive their work as less meaningful in the organizational context (Johnson & Martinez, 2024). Hence, we assume the following hypothesis:

**H2:**  *The use of ChatGPT is positively related to work engagement.*

### 1.4. Work Engagement and Job Crafting

Work engagement, which is a positive, fulfilling, work-related state of vigor, dedication, and absorption, increases employees’ proactive behaviors (Schaufeli et al., 2002). Employees who are more engaged are more likely to start job crafting, which involves self-initiated changes to tasks, relationships, and perceptions in order to enhance person–job fit (Wrzesniewski & Dutton, 2001). Research evidence indicates that job crafting leads to work engagement by increasing job resources and meaningfulness (Demerouti, 2014). The link between job crafting and work engagement is therefore two-way: engagement leads to job crafting, and job crafting, in turn, enhances engagement by increasing autonomy and motivation. A meta-analysis of longitudinal studies revealed a positive relationship between job crafting and subsequent work outcomes (Mazzetti et al., 2023), concluding that association is more robust when employees consider work as an origin of personal satisfaction. Job crafting mediates the relationship of WE with different outcomes, like flourishing and job performance (Frederick & VanderWeele, 2020). A recent study by Sharma and Nambudiri (2020) proved that engaged employees are more proactive in job crafting behavior, particularly among IT sector employees, offering an important Asian perspective on these mechanisms. A recent study by Sharma and Nambudiri (2020), conducted among IT sector employees in an Asian context, found that engaged employees were more proactive in job crafting behaviors.

In a recent study, Sharma and Nambudiri (2020) concluded that engaged employees were more proactive in job crafting behaviors among employees in the IT sector of an Asian background. In this case, the Asian view is not regarded as a homogeneous cultural group, but it is the evidence produced within a non-Western organizational context, where work relations, social expectations, and employee proactive behavior might be determined by other contextual norms than those normally studied in the Western literature.

For example, Sharma and Nambudiri (2020), in a study conducted among IT sector employees in an Asian context, found that engaged employees were more proactive in demonstrating job crafting behaviors. This evidence, drawn from a non-Western setting, further supports the positive association between work engagement and job crafting.

Consequently, we also predicted that WE would affect job crafting in the context of ChatGPT use.

**H3a:**  *Work engagement is positively related to task crafting.*

**H3b:**  *Work engagement is positively related to cognitive crafting.*

**H3c:**  *Work engagement is positively related to relational crafting.*

More precisely, increasing structural job resources and challenging demands mediates the positive influence of WE on these outcomes. A longitudinal study demonstrated that job crafting intentions indeed result in actual job crafting behaviors, while these in turn lead to higher levels of WE and improved in-role performance (Tims et al., 2015). Similarly, engagement is a powerful psychological mechanism between job crafting and employee outcomes (Schaufeli & Salanova, 2022), such as performance (Van den Heuvel et al., 2023). Furthermore, scholars argued that job crafting can be induced by technology via mechanisms through which employees’ reshape their own roles. For that reason, WE is considered as mediator between Chat GPT and job crafting.

**H4a:**  *Work engagement mediates the relationship between the use of ChatGPT and task crafting.*

**H4b:**  *Work engagement mediates the relationship between the use of ChatGPT and cognitive crafting.*

**H4c:**  *Work engagement mediates the relationship between the use of ChatGPT and relational crafting.*

### 1.5. Moderating Effect of Work-Related Curiosity

Studies depicted personal dispositions as instrumental in using AI. Kumar and Upadhyay (2022) identified a moderating role of critical thinking in the relationship between propensity to diffuse ChatGPT and customer engagement. Menon and Shilpa (2023) emphasized the factors that led to the acceptance of ChatGPT, such as perceived interactivity and privacy issues, whereas Jo (2023) affirmed the role of perceived intelligence, knowledge management, and personalization in the use of AI tools. According to these findings, the individual differences can affect the way users convert AI exposure in-to meaningful psychological outcomes. Brown and Adams (2024) expanded the concept of work-related curiosity to work engagement (WE) and its predictors. Curiosity at work is an aspect of a dispositional feature, including a propensity to pursue novelty, explore new situations that are unknown to us, and gain knowledge (Brown & Adams, 2024). As opposed to engagement, curiosity functions as a fairly constant personality attribute that defines the approach of individuals to learning and new technologies (Attridge, 2009). From a motivational perspective, curious employees are more inclined to experiment with emerging systems, test alternative prompts, and persist in learning through trial and feedback (Agarwal & Gupta, 2018). Curiosity as a depiction of intrinsic motivation tends to seek new challenges and information, as well as and deal with dynamic situations.

**Figure 1:**
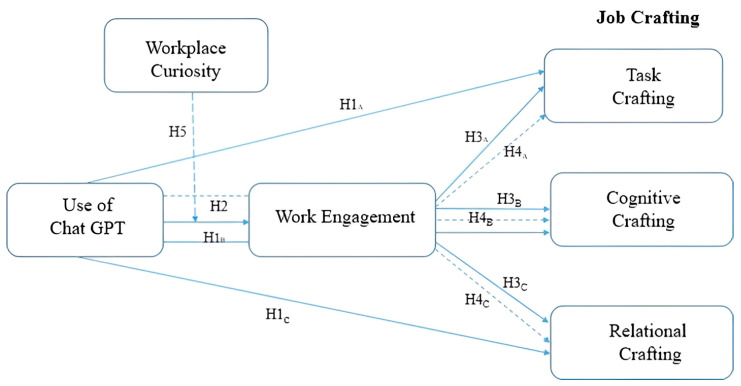
Conceptual framework.

Employees with high levels of curiosity are more likely to perceive ChatGPT as an opportunity for competence development and mastery rather than as a potential threat to their autonomy, and therefore may convert their interactions with AI into greater perceptions of resource acquisition (e.g., knowledge, confidence, and control), strengthening the effect of ChatGPT use on WE (Brown & Adams, 2024). Conversely, employees with low levels of curiosity may use ChatGPT in a more superficial or efficiency-driven manner, limiting the extent to which resource gains translate to higher engagement (Menon & Shilpa, 2023; Jo, 2023). We argue that the effects of ChatGPT use have stronger influence on work engagement in the presence of work-related curiosity. Thus, we propose that work-related curiosity moderates the relationship between ChatGPT use and WE, such that the positive association is stronger for employees with higher levels of curiosity (Agarwal & Gupta, 2018; Brown & Adams, 2024). Thus, we also predict that work-related curiosity can serve as a moderator of use of ChatGPT and WE. These relationships are depicted in Figure 1.

**H5:**  *Work-related curiosity moderates the relationship between the use of ChatGPT and work engagement.*

## 2. Materials and Methods

### 2.1. Sample and Data Collection Procedures

We collected three-wave time-lagged data from IT professionals working in IT companies in Pakistan. IT professionals possess unique characteristics because, as knowledge workers, they require an analytical mindset, professionalism, and innovativeness to succeed in their roles. It has become evident that IT professionals have been using AI tools (Speth et al., 2023) more in programming, idea generation, error fixing, comparing coding, and customization (Priestley, 2017). This study entails a three-wave time-lagged design in order to make a temporal separation of the determinants and outcomes. A minimum 15-day distance between two-time lags is enough, as recommended by Podsakoff et al. (2012, 2024). This study uses for a 2-week interval between each time lag from T1 to T3. Initially, at T1, 510 respondents were surveyed about ChatGPT use, work-related curiosity, and demographics, yielding 403 responses. In the second phase, at T2, after 2 weeks, the same respondents were surveyed on WE, with 353 responses received. Finally, at T3, which occurred after another 2 weeks, job crafting behavior was described by IT professionals, which resulted in 321 responses. After cleaning incomplete surveys, the final sample consisted of 314 usable responses for the analyses. Out of these 314 respondents, 84.8% were males, and the average age of the respondents was 22.16 years. Around 74% employees had experience of 1 to 5 years, 18% had 6 to 10 years of experience, and the remaining 8% had 11 years of experience or more in the same industry.

### 2.2. Measurements

The use of ChatGPT was measured using a 10-item scale developed by Abbas et al. (2024), through 6-point Likert-type scale ranging from 1 (“Never”) to 6 (“Always”). This scale was originally designed for students and has been slightly modified and adapted for this study.

Pilot data collection was conducted with 53 respondents to assess the questionnaire’s face validity. The scale’s validity was then assessed to ensure that it accurately captured the intended constructs in this new context. Based on the feedback from the pilot study, the questions were revised to reduce ambiguity. An example item included in the scale is “*I use ChatGPT to learn work-related concepts.*” Work-related curiosity scale was adopted from Mussel et al. (2012), which contains 10 items. A sample item includes “*I am interested in how my contribution impacts the company.*” Work engagement measurements included a nine-item scale developed by Balducci et al. (2010), containing a sample item such as “*At my work, I feel bursting with energy.*” Job crafting was measured through a 15-item scale developed by Slemp and Vella-Brodrick (2013), wherein 5 items measure each dimension of job crafting. WRC was measured using a 10-item scale (0.83) adapted from the WRC scale (Mussel et al., 2012). This curiosity scale was established initially for the use in the work context, and it is the single measure available to assess curiosity in this regard. The sample item is as follows: “*I am eager to learn.*”

### 2.3. Control Variables

Since this study’s outcome (work engagement and job crafting) may vary across demographics), ANOVA was performed to examine the significant impact of demographic variables on outcomes. The ANOVA results show that all demographic variables have insignificantly impacted job crafting and WE, which have not been controlled for further analysis.

## 3. Results

### 3.1. Data Analysis

We used partial least square structural equation modeling (PLS-SEM) to test the measurement sand structural models for the hypotheses testing (Hair & Alamer, 2022).

### 3.2. Measurement Model

All constructs were theorized in a reflective manner since the indicators are considered to be the functions of latent variables (e.g., WE was reflected by vigor, dedication, and absorption; job crafting was reflected by task, cognitive, and relational crafting). Thus, the indicators should be correlated and can be mostly interchangeable, and this is in favor of reflective specification. A formative specification was not used because the indicators do not form the constructs; instead, the constructs are assumed to cause the observed indicators, consistent with reflective measurement logic.

First, this study examined the major indicators that were mainly used to analyze the measurement model, including standardized factor loading, composite reliability (CR), Cronbach’s alpha (CA), and average variance extracted (AVE). Table 1 indicates that the measurement model meets validity and reliability criteria. The standardized facto loadings of all items in each construct were higher than the cutoff value of 0.60 (Hair et al., 2019) shown in Table 1. However, due to failure to achieve some of the recommended loading values, we considered low-loading items and dropped them only when deletion better increased the measurement quality of the construct (e.g., AVE/CR) without compromising the conceptual meaning of the scale. So, 2 out of 10 items regarding work-related curiosity and 1 out of 10 items regarding the use of ChatGPT were deleted due to low factor loading. These deletions were limited and did not alter the theoretical domain of the constructs.

The scores of composite reliability and Cronbach’s alpha for all measures exceeded the 0.7 threshold, thereby indicating construct reliability. As for PLS-SEM, CR values over 0.70 denoted good reliability of constructs that are developed (Hair et al., 2020). The AVE scores for all variables except WE were above the 0.5 threshold, indicating convergent validity (Hair et al., 2020).

**Figure 2:**
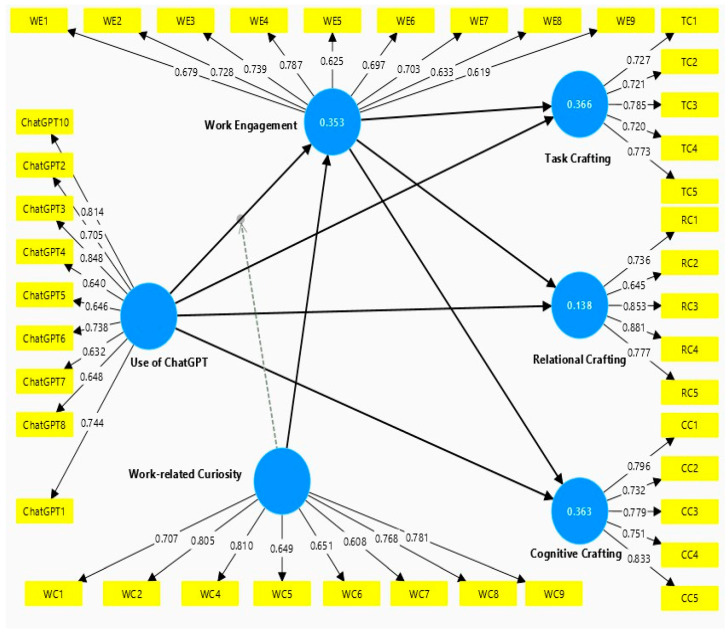
Measurement Model.

However, the AVE score for WE was 0.479. Following Fornell and Larcker’s (1981) recommendation, convergent validity may still be considered adequate when AVE is slightly below 0.50, provided that the composite reliability is sufficiently high. The composite reliability for WE was 0.868, thus establishing convergent validity. Figure 2 shows measurement model testing. Figure 2 presents measurement model assessment.

Table 1 depicts AVE values, which are acceptable in range of 0.5. Only WE shows the value of 0.479, and AVE shows the value slightly lower than 0.5, which can be explained as CR of WE is 0.868, ensuring the consistency of the scale (Fornell & Larcker, 1981). Furthermore, discriminant validity ensures that each latent construct is distinct from other constructs. As per Fornell and Larcker’s (1981) criteria, discriminant validity is established if the square root of the AVE for each construct is larger than the correlation of that construct with other constructs. Similarly, Henseler et al. (2015) consider the heterotrait– monotrait (HTMT) ratio as a better tool for establishing discriminate validity, as a large number of researchers have also used it (Jaleel & Sarmad, 2024). The HTMT values below 0.900 are considered good for establishing discriminant validity. As shown in Table 2, all HTMT values were below the threshold, thereby establishing discriminant validity among the study’s constructs. Furthermore, in order to test multicollinearity, we calculated the variance inflation factor (VIF), which should be less than 5 (Hair et al., 2020), and, for this study, the VIF scores were less than 5.

Due to the fact that the data were gathered through a self-reported survey, there is a risk of the common method bias (CMB). To minimize this threat, anonymity was realized, and respondents were told that there were no correct and incorrect answers. The checking of a single factor given by Harman did not reveal the statistical prevalence of single factor, and the value of VIFs was less than 5 (Hair et al., 2020), which means that CMB should not have influenced the findings.

### 3.3. Structural Model

**Figure 3:**
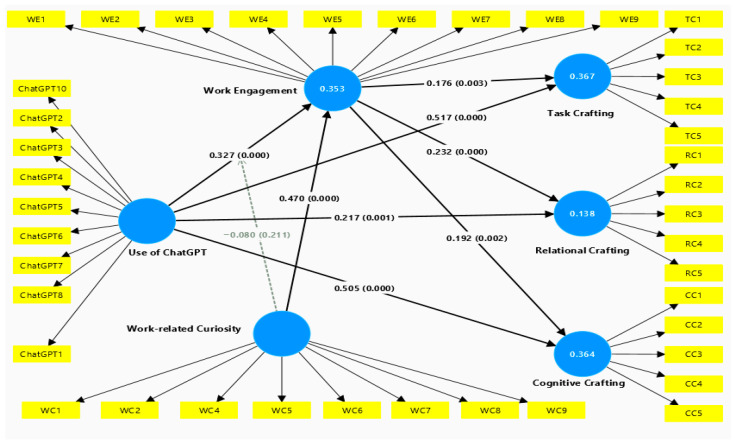
Structural model.

This study subsequently examined the hypotheses by using bootstrapping procedures with 5000 samples in SmartPLS by following instructions of Hair et al. (2020). The structural model is depicted in Figure 3. As shown in Figure 3, the hypothesized connections between the use of ChatGPT, work engagement, and job crafting dimensions are expected to be positive, contingent on their strength. As detailed in Table 3, the results showed that the use of ChatGPT was positively correlated with task crafting (β = 0.581, *p* < 0.001), cognitive crafting (β = 0.568, *p* < 0.001), and relational crafting (β = 0.293, *p* < 0.001), supporting hypotheses 1a, 1b, and 1c. It is worth noting that the greatest effects were observed on task and cognitive crafting, implying that ChatGPT primarily facilitates changes in the way employees perform and cognitively frame their work.

The results further revealed that the use of ChatGPT was positively related to work engagement (β = 0.327, *p* < 0.001). Thus, hypothesis 2 is supported, indicating that employees who frequently use ChatGPT are more highly engaged at work. Furthermore, the results showed that work engagement was positively related to task crafting (β = 0.176, *p* = 0.003), cognitive crafting (β = 0.192, *p* = 0.002), and relational crafting (β = 0.232, *p* < 0.001), supporting hypotheses 3a, 3b, and 3c. This indicates that highly engaged employees are more likely to engage in job crafting behaviors, supporting the mediating role of work engagement between the ChatGPT use and job crafting.

This study followed Preacher and Hayes’s (2004) approach for mediation analysis by performing bootstrapping to test the mediating role of work engagement between the ChatGPT use and job crafting dimensions. As expected, the results showed that work engagement significantly mediated the relationship between the ChatGPT use and task crafting (β = 0.076, *p* = 0.009), cognitive crafting (β = 0.063, *p* = 0.014), and relational crafting (β = 0.058, *p* = 0.019), supporting the mediation hypotheses. Since both the direct paths between the ChatGPT use and job crafting dimensions and the indirect effects are significant, the results indicate partial mediation. This suggests that ChatGPT has both direct and indirect effects on job crafting through increased work engagement.

Finally, Table 4 show the results of the fifth hypothesis, i.e., work-related curiosity does not moderate the relationship between the use of ChatGPT and WE, indicating that the higher work-related curiosity implies the stronger the association (β = −0.08, *p* > 0.05). It means that the relationship between the use of ChatGPT and work engagement is not significantly different at various levels of curiosity about the work. In other words, ChatGPT seems to increase the engagement irrespective of the high or low share of the employees in terms of their curiosity.

## 4. Discussion

This paper examines whether GenAI, specifically ChatGPT, drives job crafting behavior through the effect it exerts on WE. AI uses the combined effects of science and engineering to integrate machines capable of problem-solving in ways that resemble human approaches (Chikhaoui & Mehar, 2020). The findings indicate that ChatGPT positively influences all three dimensions of job crafting, both directly and indirectly, through work engagement. Work engagement partially mediates the association between the use of ChatGPT and job crafting, whereas the moderating role of work-related curiosity was not supported between the ChatGPT use and work engagement. The results are generally consistent with previous studies of Baidoo-Anu and Ansah (2023) and Vrontis et al. (2023), which suggest that ChatGPT stimulates employees’ work efficiency, imagination, and proactive behavior by providing accessible work resources in complex environments (Rasul et al., 2023). However, our study extends these works by empirically demonstrating that the impact of ChatGPT on proactive job redesign operates through a resource-based psychological mechanism—namely, work engagement. Thus, WE has been confirmed to be a significant mediator in the model, since ChatGPT is likely to enhance employees’ vigor, dedication, and absorption in tasks, which in turn increase job crafting behaviors (Sahab & Haqbeen, 2024; Agarwal & Gupta, 2018; Javaid et al., 2023). This finding supports the resource gain logic underlying COR theory, suggesting that when ChatGPT reduces cognitive strain and enhances perceived control, employees accumulate psychological resources that they reinvest in proactive work redesign.

Contrary to our expectations, work-related curiosity did not significantly moderate the relationship between the ChatGPT use and work engagement. While Brown and Adams (2024) suggested that curiosity interacts with work engagement determinants, our findings indicate that ChatGPT enhances engagement regardless of employees’ baseline curiosity levels. The interpretation here is that, as a highly accessible and user-friendly resource, ChatGPT offers resource gains that are robust enough to impact employees in general and not necessarily just the most curious workers in particular.

Meanwhile, there is still a question of excessive dependence on ChatGPT. It has been indicated as alarming in previous studies that over-reliance on AI tools can compromise independent analytical thinking and innovativeness (Dwivedi et al., 2023; Peters et al., 2023). Hence, we present positive behavioral results, but these results should be viewed in the context of these emerging concerns. According to many longitudinal studies, job crafting and WE have a positive correlation with each other and their respective outcome, including job performance (Bakker & Demerouti, 2017; Liu & Chen, 2021). When employees feel that the work is satisfying, they exhibit better connections between designing activities and achieving thriving outcomes (Frederick & VanderWeele, 2020). Job crafting behaviors aimed at increasing resources and challenging demands improve both WE and in-role performance (Tims et al., 2015). Leisure crafting also contributes to meaning and self-reflection outside work roles (Petrou et al., 2012). WE has been shown to mediate the relationship between job crafting and performance (Van den Heuvel et al., 2023). Our findings complement this stream by positioning ChatGPT as an upstream technological resource that stimulates engagement, which subsequently encourages crafting behaviors.

Research has also indicated that technology influences job crafting by enabling employees to better adjust their roles. Technology allows personalization of work, reinforcing the proactive nature of job redesign. In this respect, our study responds to recent calls to examine GenAI’s impact on employee outcomes (Brynjolfsson et al., 2023; Cardon et al., 2023; Marimon et al., 2024) by empirically linking the use of ChatGPT to behavioral work redesign through engagement. We contribute to the literature on ChatGPT and job crafting by demonstrating that engagement serves as a key explanatory mechanism. However, given the non-significant moderation finding, we refrain from concluding that curiosity systematically enhances the ChatGPT–engagement relationship. Rather, curiosity may operate through more complex or indirect pathways that warrant further investigation.

This study makes three contributions. First, it advances job crafting research by positioning ChatGPT as a distinctive AI-enabled cognitive resource that can stimulate proactive job redesign. Second, it differentiates ChatGPT from conventional digital technologies by emphasizing its generative and interactive features and by explaining why these characteristics matter for employee behavior, while extending AI research beyond adoption frameworks by examining a process model in which work engagement mediates and work-related curiosity moderates the relationship between the use of ChatGPT and job crafting. Finally, by examining three forms of job crafting in an Asian context, this study provides contextually grounded evidence on how GenAI tools shape employee behavior in emerging digital workplaces. The other theoretical contribution of this study is the analysis of three different types of job crafting in reaction to the use of ChatGPT. In terms of context, the study provides evidence relating to an Asian context, which has not been studied extensively in previous studies of AI-enabled workplace behavior. This contribution should be interpreted cautiously, as it does not assume cultural homogeneity across Asian workplaces. Instead, it implies that contextual variations in the work practices and conditions of technology adoption might determine the way in which employees are interacting with AI resources like ChatGPT.

In practice, the results suggest that ChatGPT can be used as a supporting digital resource that promotes active work behaviors. Nevertheless, managers are not to treat GenAI tools as solutions that should be embraced without question. Instead, they ought to focus on their organized, conscientious, and responsible applications in organizational contexts. In this regard, IT professionals working with ChatGPT might be required to show tangible performance and resiliency enhancements to ensure long-term organizational support (Katar, 2020). It is necessary to encourage managers to enhance long-term industry employee competence regarding AI applications instead of concentrating on short-term efficiency benefits.

The results also indicate that ChatGPT can contribute positively to employee outcomes in terms of task, cognitive, and relational crafting. Yet, such findings cannot be viewed as proof of the ability of AI to enhance every aspect of employee functioning. Companies, and especially IT companies might, therefore, find it beneficial to offer training on how to effectively, ethically, and responsibly use AI to achieve the most benefits and minimize potential risks. Generally, ChatGPT seems to be able to decrease cognitive load and certain everyday job requirements, although its usefulness relies on the extent to which it be implemented into larger organizations and workflows

### Limitations and Future Research

The use of technology, such as GenAI, contributes to job crafting, as disclosed by this study. Despite various valuable realizations, associated limitations also exist. The cross-sectional design employed in time-lagged data collection is itself a unique study design. It limits the effects of time, as observed in longitudinal studies. Future research should be focused on understanding the long-term effects of ChatGPT on the cognition of employees, and these efforts can explore subsequent effects of this technology on cognition, as well as on employees’ behavior. Leisure crafting can be used to take time off from the working roles outside the workplace to foster meaning and self-reflection (Petrou et al., 2012). WE forms the mediator between job crafting and performance, thus creating the connection in the workplace between proactive behaviors and outcomes, like those found by few authors (Van den Heuvel et al., 2023). Despite the positive implications of the usage of ChatGPT, this paper also recognizes a few potential challenges. Since the literature depicted work engagement as an outcome of job crafting and vice versa, this study only focused on one direction—work engagement leads to job crafting. Hence, this approach can be acknowledged as a limitation of the study, and future studies may explore reciprocal relationships under JD-R. There is a concern, according to Abbas et al. (2024), regarding AI’s effects on creativity and critical thinking when using ChatGPT. Thus, future studies can explore the cognitive factors that can translate into behavioral outcomes. Similarly, the potential damage to deeper cognitive functions—the ability to think more critically—needs to be addressed. Peters et al. (2023) and Dwivedi et al. (2023) specifically posit that employees will not be able to think more critically nor informatively due to over-reliance on AI tools. Future studies should build on these possible negative ramifications to paint a comprehensive view of the impact that ChatGPT could have on performance at workplaces. Furthermore, future research should assess the additional boundary conditions that could make AI effective—including personality traits and organizational support. Such knowledge will feed into developing a strategy to exploit AI technologies in ways that produce maximum benefit while minimizing the risks associated with their use in the workplace. The majority of respondents in this study were male, and this might posit a gender bias, even though the majority of workers in this industry are males. Therefore, we suggest that future studies investigate gender as a moderator between using AI and job crafting. Additionally, the average age of respondents was 22.16 years, which indicates that IT firms employ a majority of early-career workers who use AI in their jobs. Future studies may capture all career stages to justify the spread of use of AI in the workplace.

## 5. Conclusions

This study highlights the role of generative AI, particularly ChatGPT, in shaping employee behavior in Pakistan’s IT sector. Grounded in the Conservation of Resources (COR) theory, the findings suggest that ChatGPT can function as a digital resource that supports employees’ resource gain (e.g., reduced cognitive strain and enhanced perceived control). In line with this logic, the ChatGPT use is associated with higher work engagement (WE), which is linked to greater job crafting across task, cognitive, and relational dimensions.

The findings demonstrate that WE is a valuable mediator between the use of ChatGPT and job crafting. Simultaneously, these findings must be viewed with reservations due to the design and context of the study. Although the responsible use of GenAI tools can lead to positive work outcomes, business organizations must not expect equal benefits for employees or work, and they must be vigilant to risks such as excessive dependence on automation. In practice, the research implies that the implementation of AI should be perceived by managers and policymakers as a socio-technical change instead of a technical upgrade. Instead of working solely on the adoption, organizations may prioritize guidance and training to motivate employees to adopt AI as a learning, problem-solving, and workflow-enhancing tool. Well-managed and appropriately tempered approaches can contribute to creating more involved and versatile working teams as GenAI applications become increasingly widespread.

## Figures and Tables

**Table 1:** Factor loading, reliability, and validity.

Items AVE	Loadings	CA	CR
Use of ChatGPT	Above 0.6	0.879	0.885
0.514			
Work-related curiosity	Above 0.6	0.872	0.901
0.527			
Work engagement	Above 0.6	0.863	0.868
0.479			
Task crafting	Above 0.6	0.837	0.846
0.527			
Cognitive crafting	Above 0.6	0.800	0.804
0.556			
Relational crafting	Above 0.6	0.841	0.802
0.613			

**Table 2:** Discriminant validity (Fornell–Larcker criterion).

Constructs	1	2	3	4	5	6
1. Use of GenAI (ChatGPT)	**0.717**					
2. Work-related curiosity	0.079	**0.726**				
3. Work engagement	0.374	0.483	**0.692**			
4. Task crafting	0.304	0.208	0.312	**0.733**		
5. Relational crafting	0.575	0.095	0.382	0.601	**0.746**	
6. Cognitive crafting	0.583	0.142	0.370	0.544	0.711	**0.779**

Note: diagonal values (bold) indicate the square root of AVE for each construct.

**Table 3:** Hypothesis testing.

Paths	*Β*	SE	*t*-Value	*p*-Value		
Use of ChatGPT → TC	0.581	0.053	10.842	<0.001		
Use of ChatGPT → CC	0.568	0.056	10.882	<0.001		
Use of ChatGPT → RC	0.293	0.063	4.399	<0.001		
Use of ChatGPT → WC	0.327	0.065	5.024	<0.001		
WC → TC	0.176	0.060	5.024	0.003		
WC → CC	0.192	0.063	3.028	0.002		
WC → RC	0.232	0.066	3.509	<0.001		
Use of ChatGPT → WC → TC	0.517	0.057	9.064	<0.001		
Use of ChatGPT → WC → CC	0.505	0.060	8.451	<0.001		
Use of ChatGPT → WC → RC	0.217	0.065	3.349	0.001		
					**LL**	**UL**
**Bootstrap Results**					**95% CI**	**95% CI**
Indirect effect of the use of ChatGPT → TC	0.076	0.029	2.605	0.009	0.016	0.109
Indirect effect of the use of ChatGPT → CC	0.063	0.025	2.470	0.014	0.018	0.117
Indirect effect of the use of ChatGPT → RC	0.058	0.025	2.337	0.019	0.026	0.149

Note: WC = work-related curiosity; TC = task crafting; CC = cognitive crafting; RC = relational crafting; CI = confidence interval; UL = upper limit; LL = lower limit; SE = Standard Error.

**Table 4:** Moderation of work-related curiosity.

Paths	B	SE	*t*-Value	*p*-Value	LL 95% CI	UL 95% CI
Use of ChatGPT × WC → WE	−0.080	0.064	1.251	0.211	−0.206	0.104

Note: WC = work-related curiosity; WE = work engagement.

## Data Availability

The data presented in this study is available on request from the corresponding author.
